# The PINK1-Mediated Crosstalk between Neural Cells and the Underlying Link to Parkinson’s Disease

**DOI:** 10.3390/cells10061395

**Published:** 2021-06-05

**Authors:** Elvira Pequeno Leites, Vanessa Alexandra Morais

**Affiliations:** Instituto de Medicina Molecular-João Lobo Antunes, Faculdade de Lisboa, Universidade de Lisboa, 1649-028 Lisbon, Portugal; elviraleites@medicina.ulisboa.pt

**Keywords:** Parkinson’s disease, mitochondrial dysfunction, PINK1, neurons, astrocytes, microglia

## Abstract

Mitochondrial dysfunction has a fundamental role in the development of idiopathic and familiar forms of Parkinson’s disease (PD). The nuclear-encoded mitochondrial kinase PINK1, linked to familial PD, is responsible for diverse mechanisms of mitochondrial quality control, ATP production, mitochondrial-mediated apoptosis and neuroinflammation. The main pathological hallmark of PD is the loss of dopaminergic neurons. However, novel discoveries have brought forward the concept that a disruption in overall brain homeostasis may be the underlying cause of this neurodegeneration disease. To sustain this, astrocytes and microglia cells lacking PINK1 have revealed increased neuroinflammation and deficits in physiological roles, such as decreased wound healing capacity and ATP production, which clearly indicate involvement of these cells in the physiopathology of PD. PINK1 executes vital functions within mitochondrial regulation that have a detrimental impact on the development and progression of PD. Hence, in this review, we aim to broaden the horizon of PINK1-mediated phenotypes occurring in neurons, astrocytes and microglia and, ultimately, highlight the importance of the crosstalk between these neural cells that is crucial for brain homeostasis.

## 1. Introduction

### 1.1. Parkinson’s Disease

Parkinson’s disease (PD) is a progressive neurodegenerative movement disorder, mainly characterized by the loss of dopaminergic neurons and the presence of Lewy bodies [1]. Environmental and genetic factors are high contributors to the appearance of this disorder [2]. Non-genetic risk factors include aging, life habits such as smoking, drug abuse and exposure to pesticides, herbicides, and heavy metals [2]. However, a recent study demonstrated that smoking could be a protective factor of PD [3]. On the other hand, genetic mutations have been linked to PD and explain several of the features associated with this pathology [4]. Some of the most prevalently PD-linked mutations are encountered in the genes encoding for α-synuclein (SNCA), leucine-rich repeat kinase 2 (LRRK2), Parkin, PTEN-induced putative kinase (PINK1) and DJ-1 [1].

### 1.2. Mitochondria and Their Role in PD

Without a doubt, mitochondrial function is crucial for well-being. Hence, the mal-function of this organelle appears associated with multiple diseases, from neurodegeneration to muscle degeneration, among many more [5]. Additionally to the conventional role of ATP production, mitochondria are responsible for calcium homeostasis, apoptosis and reactive oxygen species (ROS) production [6]. All mitochondria-mediated functions are regulated through well-synchronized pathways. These pathways go from mitochondrial dynamics, such as fusion and fission, transport and arrest, all the way to mitochondrial morphology and cristae remodeling. Furthermore, within these synchronized pathways, the formation of mitochondrial-derived vesicles (MDVs) and mitochondria clearance, also known as mitophagy [6]. Knowing that mitochondria are directly associated to these vital cellular processes, it comes as no surprise the impact that their malfunction may have upon cell survival.

The brain is mainly constituted by neurons, astrocytes, microglia and oligodendrocytes. These neural cells have different functions and requirements. Thus one would suspect that mitochondria also would have defined roles for each of these cell types depending on their necessities [7]. In neurons, it has been shown that mitochondria are important for axonal development and regeneration, as they fulfill the local ATP and calcium requirements [7]. These two functions are also required to support synaptic function and plasticity, where ATP and calcium are needed for synaptic-vesicles pool formation and release, respectively [7]. On the other hand, mitochondria found in astrocytes have been shown to be key in regulating glutamate transporters [8]. Astrocytes mainly rely on ATP produced by aerobic glycolysis rather than by oxidative phosphorylation [8]. This cell type is able to produce lactate from pyruvate under aerobic conditions, which may be transmitted to other cells and be used to initiate different pathways such as mitochondrial respiration [9]. The release of lactate from the astrocytes into the extracellular space and its consequent uptake by neurons to perform oxidative phosphorylation, forming the neuron-astrocyte lactate shuttle, further highlights the importance of astrocytes to neuron function [10]. Astrocytes are also able to convert pyruvate into oxaloacetate, allowing the entrance of pyruvate into mitochondria in the absence of α-ketoglutarate [8,11]. This conversion, performed by pyruvate carboxylase, an enzyme highly enriched in astrocytes, will allow the formation of glutamate and eventually, glutamine, which is fundamental for the glutamine-glutamate cycling between neurons and astrocytes [11,12]. Since the transport of glutamate and its precursor glutamine from the blood to the brain is a rather slow process [13], glutamate, an excitatory transmitter, and its decarboxylation product γ-aminobutyric acid (GABA), an inhibitory transmitter, need to be synthesized by neural cells [14]. However, neurons are not able to produce glutamate as they lack the α-ketoglutarate enzyme [14]. Therefore, astrocytes that have high pyruvate carboxylase activity are able to produce a higher amount of oxaloacetate, leading to the formation of more α-ketoglutarate, thus more glutamate [11,15]. This glutamate, together with the neuronal-released glutamate, is metabolized by an astrocyte-specific enzyme called glutamine synthetase, leading to the formation of glutamine, which is transported to neurons and converted into glutamate or GABA, according to the necessity of the neurons [14]. The uptake of glutamate by astrocytes is made via EAATs (excitatory amino acid transports) together with sodium ions, which are then excreted by the action of the Na^+^/K^+^ ATPase expending ATP [16]. This reaction, mediated by Na^+^/K^+^ ATPase, leads to glucose uptake from the circulation through the glucose transporter GLUT1, which will be converted into lactate and shuttled to neurons to be used as an energy substrate [16]. A proteomic study, using an engineered MitoTag mouse, revealed that astrocytes had increased expression levels of peroxisomal proteins and enzymes involved in mitochondrial β-oxidation when compared with Purkinje cells and granule cells [17]. Regarding microglia, not much is known about the specifications of mitochondria in these cells in resting conditions. However, when comparing activated with non-activated microglia, studies have revealed that after the activation of microglia, there is a switch from mitochondrial oxidative phosphorylation to anaerobic glycolysis [8,18]. Also, inhibition of Complex I leads to activation of microglia, while deficits in mitochondrial fission pathways reduce activation, showing the importance of mitochondria dynamics in the activation status of the cell [19,20].

Notably, mitochondria are crucial for mediating cell survival and ultimately tissue or organ homeostasis. Therefore, it is not surprising that mitochondrial dysfunction is implicated in several diseases, namely brain-related disorders. For instance, the development of Parkinson-like symptoms has been associated to close and prolonged exposure to pesticides, herbicides and neurotoxins such as MPTP [2]. Rotenone and paraquat are two commonly used pesticides that are known mitochondrial toxins and that lead to dopaminergic neuron loss [21]. Rotenone, an inhibitor of mitochondrial Complex I, and paraquat, which prevents electron transfer to NADPH, were shown to cause oxidative stress by triggering intracellular ROS formation in the striatum [21,22]. Although no direct connection of rotenone and paraquat with PD patients was proven, a study using two groups of pesticides, which inhibit Complex I and increase oxidative stress, showed that prolonged use of these compounds has a positive correlation with the development of PD [21]. MPP^+^, a metabolite of MPTP, enters dopaminergic neurons inhibiting mitochondrial respiration by inhibiting Complex I [23]. These studies show that mitochondrial dysfunction can, in principle, be one of the underlying causes of PD.

The identification of mutations in genes that encode Parkin, PINK1 and even mutations in mitochondrial DNA further strengthens the hypothesis that mitochondria are one of the main causes of PD [5]. When studying early-stage PD patients and pre-symptomatic PD patients, which are represented by incidental Lewy body disease cases, mitochondrial DNA (mtDNA) mutations were observed in *substantia nigra* neurons when compared to control samples [24]. Also, when comparing total mtDNA deletions/rearrangements of patients with PD, multiple system atrophy (MSA), dementia with Lewy bodies (DLB), Alzheimer disease (AD) and age-matched controls, the number and variety of mtDNA rearrangements were significantly increased in PD patients’ brains [25]. Loss-of-function mutations in Parkin and PINK1 are related to alterations in mitochondrial function either by impairing Ca^2+^ homeostasis and ATP production, by impairing the clearance of damaged mitochondria in a process called mitophagy, by increasing cell apoptosis in a mitochondrial-dependent manner, among a variety of other pathways that are impaired when PINK1 is mutated [26,27,28,29].

PINK1, a nuclear-encoded mitochondrial serine/threonine kinase, is a promiscuous kinase as the kinases’ substrates are phosphorylated depending on the overall status of the mitochondria [30]. Under healthy conditions, PINK1 is recruited into the mitochondria, where it is cleaved by different proteases [31]. Primarily, PINK1 is cleaved by the mitochondrial processing peptidase (MPP), followed by presenilin-associated rhomboid-like protease (PARL), m-AAA and ClXP [32]. These cleavages mediate the turnover of PINK1, ending with the retro-translocation of PINK1 to the cytosol, where further processing occurs in a proteasome-dependent manner [33]. During the internalization of PINK1 into the mitochondria, proteins such as NDUFA10, TRAP1 (TNF receptor-associated protein 1), BCL-xL and HtrA2 are phosphorylated [34,35,36,37]. NDUFA10 is a Complex I subunit, and its phosphorylation mediates the overall enzymatic function of the ETC, and so it is important for ATP production [38]. Acting as a cell survival mechanism, PINK1 phosphorylates BCL-xL inhibiting in pro-apoptotic cleavage [37]. On the other hand, phosphorylation of mitochondrial chaperone TRAP1 and HtrA2 protects cells from mitochondrial-induced apoptosis [35,36]. However, when PINK1 encounters unhealthy mitochondria, the accumulation of full-length PINK1 occurs at the outer mitochondrial membrane, leading to an increase in the recruitment of cytosolic Parkin, followed by the PINK1-mediated phosphorylation of Parkin, ubiquitin and PINK1 itself and giving rise to mitophagy, a mitochondrial-specific clearance pathway [39,40,41]. Parkin, also a known PINK1 substrate, is a cytosolic ubiquitin E3 ligase known to cause PD [42]. Additionally, when mitochondria contain damaged cargo, this PINK1-Parkin interaction is responsible for the formation of mitochondrial-derived vesicles that were shown to be a delivery mechanism of oxidized cargo to the late endosome [43,44]. The formation of these vesicles depends on the presence of PINK1 and Parkin, and it is a process that precedes mitophagy, indicating that it is a first attempt to rescue mitochondria before initiating mitophagy [43]. The triggering of MDVs also differs from mitophagy. While mitophagy requires a global mitochondrial depolarization, MDVs can be generated with the increase in ROS [43]. However, the molecular mechanism by which mitochondrial-derived vesicles are formed is still not well known.

Although these studies postulate that mitochondrial dysfunction occurs in several forms of PD, the fact that these dysfunctions mainly afflict dopaminergic neurons needs to be clarified.

## 2. Neurons in PD

**Figure 1:**
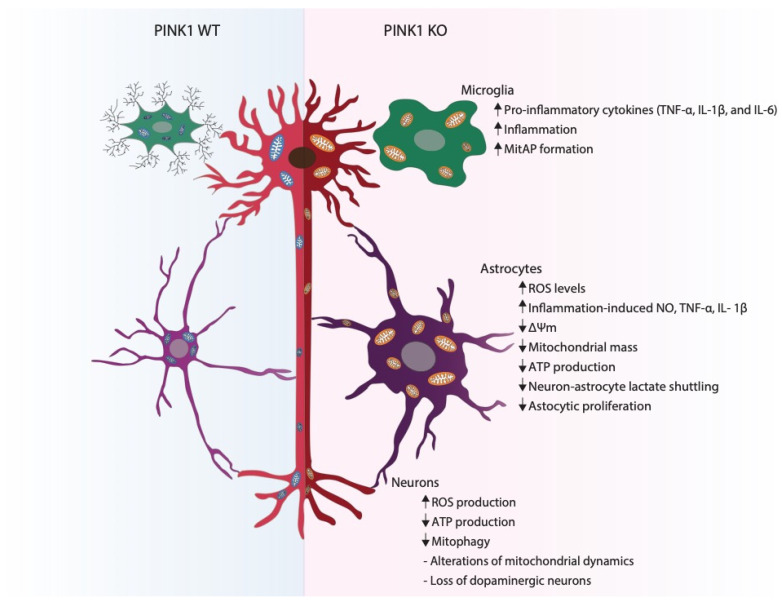
Impact of PINK1 deficiency in neural cells. When compared with PINK1 WT, PINK1 KO microglia shows an increase in the pro-inflammatory release of the cytokines TNF-α, IL-1β and IL-6, consequently leading to an increase in overall brain inflammation. Additionally, in the absence of PINK1, an increase in mitochondrial antigen presentation (MitAP) occurs, indicating an activation of autoimmune mechanisms. In astrocytes lacking PINK1, an increase in reactive oxygen species (ROS), inflammation-induced nitric oxide (NO) levels and TNF-α and IL-1β production has been observed, while a decrease in mitochondrial membrane potential (Δψm), mitochondrial mass, ATP production and glucose-uptake capacity occurs. These alterations prime a decreased astrocytic proliferation ability. In neurons, PINK1 loss-of-function leads to a decrease in ATP production and mitochondrial clearance, as well as an increase in ROS production. Absence of PINK1 also alters mitochondrial dynamics. All these alterations lead to an ultimate loss of dopaminergic neurons.

Dopaminergic neurons are particularly sensitive to the changes that happen in a PD-afflicted brain, and a progressive malfunction and eventual loss of these neurons lead to the appearance of motor symptoms [1]. Axon length and the level of myelinization are plausible reasons why some neurons are more predisposed to enter apoptosis than others [45]. Other than the fact that the more afflicted neurons have long and thin axons, they are also unmyelinated or only partially myelinated, as previously shown by Braak [45,46]. This could be explained by their extremely high energy turnover and possible consequent exposure to oxidative stress [45]. However, this increase in susceptibility is still not well known. Studies performed in *drosophila* showed that dopaminergic neurons appear more sensitive to oxidative stress, a phenotype that was reverted with the use of antioxidants [47]. Additionally, PINK1 loss-of-function was shown to be implicated in this progressive loss of dopaminergic neurons in *drosophila* as it leads to increasing levels of oxidative stress [47]. Mutations in the *PINK1* gene were associated with familial and sporadic early-onset PD [48,49]. Although genetic mutations are mostly associated with familial forms of PD, *PINK1* mutations were found in an Italian cohort of sporadic patients [49]. However, the *PINK1* gene is not the only PD-related gene associated with sporadic Parkinson’s disease. PARKIN and DJ-1 have also been associated with this form of the disorder [49]. For these reasons, it is important to study the impact of PINK1 in neurons under physiological and pathological conditions in order to fully understand how this mitochondrial kinase impacts neuronal loss (Figure 1).

### PINK1 in Neurons

PINK1 is a key regulator of mitochondrial quality control. When PINK1 is mutated and is not able to perform its functions in a healthy mitochondrion, ATP production decreases, ROS production increases, increasing neurotoxicity and dopaminergic neuronal death either by the increase of ROS or by the absence of protective pathways [50]. The impact of increased ROS is supported by the protection of dopaminergic neurons when using antioxidants in PINK1-dependent models [47]. The impact of PINK1 downregulation in *drosophila* was accessed by comparing dopaminergic and serotonergic neurons, and only dopaminergic neurons suffered progressive neurodegeneration [47]. However, dopaminergic neurons were not the only cells affected, as an ommatidial degeneration was also observed [47]. The reason why dopaminergic are more sensitive than other neuronal types to the lack of PINK1 is still an unanswered question.

When mitochondria are damaged, and in order to inhibit its movement to an energy-dependent region in neurons, Miro (mitochondria Rho) is phosphorylated by PINK,1 leading to a mitochondrial arrest [51]. Miro was suggested to be an important adaptor for the crosstalk between dynein and kinesin transport, mediating the anterograde and retrograde transport of mitochondria in neurons [51]. However, when mitochondria are damaged, Miro appears to be phosphorylated at Ser^156^ by PINK1 and ubiquitinated by Parkin, inhibiting its action and consequent mitochondrial movement [51,52]. In this situation, fusion should be decreased and fission increased in order to degrade the minimum amount of mitochondria necessary to eliminate the damage. For this, when mitochondria are depolarized, Mfn2 (mitofusin 2) and DRP1 (dynamin-related protein 1), proteins involved in mitochondrial fusion and fission, respectively, are phosphorylated in a PINK1-dependent manner, highlighting the importance of PINK1 in regulating this process [53,54,55]. Mfn2 is one of the proteins responsible for the fusion of mitochondria. However, when Mfn2 is phosphorylated by PINK1 and consequently ubiquitinated by Parkin, it is degraded, preventing a fusion event [53]. In the case of DRP1, a key player of mitochondrial fission, when phosphorylated at Ser^616^ in a PINK1-dependent fashion, fission is promoted. However, no mechanistic insights are known [56]. In PINK1-linked PD, this control and clearance of damaged mitochondria, as well as this fusion and fission balance, is impaired, leading to the release of ROS and damaged mitochondrial DNA, both of which are toxic products that increase neurotoxicity and afflict dopaminergic neurons [50,57].

PINK1 and Parkin were shown to regulate mitochondrial biogenesis and to maintain a pool of healthy mitochondria in dopaminergic neurons through the PARIS/PGC-1α axis [58]. However, when PINK1 or Parkin are defective, a progressive dopaminergic neuron loss occurs, demonstrating another pathway where PINK1’s loss-of-function could be the cause of PD [57,58].

As previously mentioned, mitochondria are responsible for calcium homeostasis, and PINK1 also regulates this mechanism, as proven by the impairment of mitochondrial calcium efflux and consequent mitochondrial calcium overload in the absence of PINK1 [59]. This calcium dysregulation results in increased ROS levels in PINK1 KO mouse neurons, leading to an impaired respiration and mitochondrial permeability transition pore (PTP) opening, ultimately promoting neuronal death [59]. This is of particular importance for neurons as they are more susceptible to calcium influxes and increased oxidative stress, as in the case of neurons from the *substantia nigra* [59].

The loss of dopaminergic neurons is a pathological hallmark of PD. However, even though PINK1 is present in all cells of the body, only the neurons from PD patients are afflicted [60]. When looking into PINK1 function in other organs, such as kidney, PINK1-mediated mitophagy has a protective role, preventing renal tubular epithelial cells apoptosis and tissue damage in contrast-induced acute kidney injury by reducing mitochondrial ROS and neutrophil/lymphocyte ratio family pyrin domain containing 3 (NLRP3) inflammasome activation, in mice [61]. In mice livers, PINK1-mediated mitophagy was shown to have a protective role against non-alcoholic fatty liver disease (NAFLD) by clearing damaged mitochondria and allowing cyanidin-3-O-glucoside (C3G) to suppress oxidative stress, NLRP3 inflammasome activation and improving glucose metabolism [62]. In adult mouse cardiomyocytes, phosphorylation of PINK1 at Ser^495^, by AMP-activated protein kinase α2 (AMPKα2), was shown to increase mitophagy after stimulation, decreasing ROS production and apoptosis of cardiomyocytes demonstrating a role in preventing the progression of heart failure [63]. Taking these protective roles in different organs and diseases is not surprising that according to different insults and different environments, PINK1 has different functions and significance. The increase in sensitivity of dopaminergic neurons to the absence of PINK1 is still not known. However, one could argue that instead of being more sensitive to the absence of PINK1, these neurons could be more sensitive to changes in their environment that are caused by the lack of PINK1-mediated mitochondrial quality control. While PD patients age, they are exposed to different diseases, such as bacterial or viral infections. These changes in the body’s immunity could be an explanation to why PD patients develop symptoms after some years, even when they have PINK1 mutations since birth, as shown in the study where a bacterial infection was enough to induce PD-like symptoms in mice lacking PINK1 [64]. With this stimulus, microglia and astrocytes lacking PINK1 may not be able to restore their physiological function and support neurons. For these reasons, and as neurons are sensitive to a homeostatic environment in order to maintain their function and plasticity, it is important to decipher the impact that PINK1 loss-of-function causes in different cell types.

## 3. Astrocytes in PD

Neurons need to be in contact with functioning astrocytes in order to maintain synaptic homeostasis, local blood flow and neural network activity [65]. Astrocytes are the most populous sub-type of glial cells in the brain [66]. In addition to the main functions already mentioned above, the importance of astrocyte to dopaminergic neurons survival was further underlined when the protective function of GDNF (glial-derived neurotrophic factor), one of the neurotrophic molecules released by astrocytes, was observed [67]. Neuroinflammation is a well-demonstrated characteristic of PD [68]. This process can be mediated either by the activation of astrocytes or microglia [69,70]. Results obtained using PD-patient iPSC-derived astrocytes showed that α-synuclein also accumulates in these cells leading to an impairment in chaperone-mediated autophagy that increases the accumulation of α-synuclein resulting in non-cell-autonomous neurodegeneration [71]. This ability of astrocytes to uptake α-synuclein, decreasing its toxicity towards neurons, leads to an increase in intracellular toxic deposits of α-synuclein in astrocytes, consequently resulting in mitochondrial damage reflected by the presence of fragmented mitochondria and an overall decrease in ATP content [72]. Astrocytes also have the ability to keep neuronal homeostasis by taking up cellular debris or other toxic material that can be released from neighboring cells [73]. This feature is also important at the beginning of PD as it will reduce inflammation and also during the development of the disease as the death of dopaminergic neurons occurs. Recently, it was shown that astrocytes have the capacity to degrade dysfunctional mitochondria that originated from afflicted dopaminergic neurons, and consequently by providing healthy mitochondria to neurons, revealing once again the importance of neuron-astrocyte communication [74,75].

Furthermore, since astrocytes provide energy to neurons, mitochondrial dysfunction can also have a major impact in neuronal survival [8]. In accordance with this fact, PINK1 loss-of-function has started to be investigated in astrocytes.

### PINK1 in Astrocytes

It has been reported that PINK1 expression increases in astrocytes during mouse brain development and that PINK1 levels can affect the number of glial fibrillary acidic protein (GFAP)-positive astrocytes, GFAP being a widely-used protein maker for astrocytes [76]. Choi and co-workers conclude that PINK1 is a crucial protein for the development and function of astrocytes. However, the molecular mechanism remains elusive (Figure 1) [76]. As previously mentioned, in the presence of dysfunctional mitochondria, PINK1 phosphorylates Parkin and ubiquitin [40]. Even though ubiquitin phosphorylation by PINK1 is increased in astrocytes, when compared with other neural types, the physiological explanation for this event is not yet known [77]. Since PINK1 is so important for maintaining a healthy pool of mitochondria, it is not surprising that its absence in astrocytes leads to mitochondrial defects, such as decreased mitochondrial membrane potential, mitochondrial mass, increased ROS levels, decreased ATP production and decreased glucose-uptake ability [78]. All these mitochondrial phenotypes lead to a decreased proliferation of astrocytes, consequently leading to decreased wound healing capacity, as well as all other basal functions of astrocytes [78]. In the absence of PINK1, astrocytes stimulated with lipopolysaccharide (LPS) and interferon-γ (IFN-γ), present an abnormal innate immune response and increased inflammation-induced nitric oxide (NO), tumor necrosis factor-alpha (TNF-α) and interleukin-1 β (IL-1β) production, a possible mechanism through which neurons die [79].

Although astrocytes mediate inflammation and could, in principle, be responsible for a neurotoxic effect, microglia can also regulate and activate astrocytes by releasing soluble cytokines and chemokines [80]. The mitochondrial-mediated activation of astrocytes can be done by increasing the release of TNF-α and IL-1β by microglia, inducing morphological and biochemical alterations in astrocytes [81]. Having this in mind, microglia is another highly relevant cell type in PINK1-dependent PD.

## 4. Microglia in PD

Defined as the residing macrophages of the central nervous system, microglia are the most abundant immune cells in the brain [82]. The main function of microglia is to protect the brain from injury [83]. Microglia have to be able to regulate the inflammation either through repair, regeneration or cytotoxicity [83]. Depending on the activation state of microglia, these can either release pro-inflammatory cytokines or neurotoxic molecules that can potentiate the inflammation, being cytotoxic, or produce anti-inflammatory molecules, neurotrophic factors or increasing their engulfment capacity that help to restore homeostasis, promoting repair or regeneration [83]. Although microglia is mainly associated with inflammation, it was shown that in multiple sclerosis, it has an important role in promoting tissue recovery, either by producing protective factors for remyelination, phagocyting apoptotic cells and debris promoting regeneration and proliferation of stem cells, or recruiting oligodendrocytes precursors cells stimulating neurogenesis [84]. A variety of factors, such as duration of the insult, type of insult, interaction with other cell types and even the amount of cytokines released by microglia, will determine if the action of microglia is beneficial or harmful for the brain, and this will be the difference between restoring the homeostasis of the brain or supporting the progression of neurodegenerative disease [83]. Recently more importance has been given to microglia, and besides their pro-inflammatory role, these cells are also able to engulf debris and release anti-inflammatory factors, such as transforming growth factor (TGF)-β or IL-10 [82]. In PD, dopaminergic neurons release aggregates of α-synuclein when entering apoptosis that triggers a microglia-mediated pro-inflammatory behavior [85]. Under physiological conditions, microglia are responsible for synaptic pruning and remodeling, engulfing apoptotic cells and cell debris [86,87].

PET (positron emission tomography) studies performed using PD patients, demonstrate that microglia activation is an early and prolonged response of PD [88]. Also, neuroinflammation mediated by IL-1β, which is released by microglia and can activate astrocytes, increases dopamine neurons’ susceptibility to death [70]. On the other hand, inhibition of astrocytic activation by microglia is neuroprotective in PD models [89].

### PINK1 in Microglia

Since microglia have pro and anti-inflammatory functions, it is important to know what happens to this cell type when in the presence of mutated PINK1 (Figure 1).

In PD, it was shown that aggregates of α-synuclein result in reactive pro-inflammatory microglia leading to an increase in TNF-α, NO, and IL-1β [90]. However, in the absence of PINK1, an increase in pro-inflammatory released cytokines (TNF-α, IL-1β, and IL-6) in injured mouse brain slices is observed, suggesting that PINK1 has a protective role [91]. A few years ago, it was shown that PINK1 and Parkin have an important role in adaptive immunity through the repression of MitAP (mitochondrial antigen presentation) [92]. This process occurs in immune cells, and is stimulated by inflammatory conditions, suggesting that PINK1 also has an impact on immunity [92]. After this discovery, it was shown that a Gram-negative bacterial infection in the intestines of PINK1 knock-out mice increases MitAP and autoimmune mechanisms leading to a decrease in dopaminergic neuron density [64]. Since microglia are the resident macrophages of the brain and inflammation is a marked feature of PD, the impact that PINK1′s loss-of-function in microglia needs to be clarified. However, the previously described functions of PINK1 acting as a mitochondrial quality control regulator should not be discarded when considering the overall well-being of microglia and the crucial role at keeping dopaminergic neurons healthy and in a low inflammatory environment.

## 5. PINK1, a Putative Mediator of the Crosstalk between Neural Cells

Even though there are other therapeutic approaches under development and continuous clinical trials ongoing, such as gene therapies, immunotherapies targeting α-synuclein, or stem cell-based treatments, levodopa is at present the most commonly used treatment for PD patients as it significantly reduces motor symptoms [93,94]. However, and in order to develop novel treatments for PD, it is important to decipher the molecular mechanisms responsible for neuronal death and ultimately disease progression by taking into account overall brain homeostasis. Crosstalk between neurons, astrocytes and microglia is becoming more evident. The sensitivity of dopaminergic neurons to impaired environmental homeostasis appears as one of the main causes of PD, the maintenance of this homeostasis is the responsibility of the astrocytes and the microglia [1]. PINK1 is a key player for maintaining mitochondrial fitness [6]. For this reason, it is crucial to unravel the specific impact that PINK1 mutations have upon these three neural cell types.

In this review, we describe different phenotypes mediated by the absence of PINK1 that, independently of its localization, be it in neurons, astrocytes or microglia, lead to PD. The homeostasis required for an adequate function and survival of neurons is disrupted when PINK1 is not able to perform properly in astrocytes or microglia. An increase in inflammation, mediated by microglia and astrocytes, for a long period of time is not beneficial for neuron survival, ultimately leading to neuronal dysfunction and death [79,88]. The decreased ATP production by astrocytes lacking PINK1 could potentially affect the overall energy levels of neurons, resulting in neuronal deficit and increased ROS production, which will activate microglia and initiate an inflammatory response. Additionally, impaired PINK1 present in astrocytes may reduce the ability of astrocytic-mitochondrial transfer to damaged neurons, leading to an accumulation of damaged mitochondria in these cells. Baring this crosstalk between neuron-microglia-astrocyte in mind, the impact of PINK1 loss-of-function is detrimental to maintain a healthy pool of mitochondria within each neural cell type and, ultimately, to regulate efficient and robust bioenergetics crosstalk between these cells.

## 6. Conclusions

Although dopaminergic neurons are the most affected cells in PD, it has been recently demonstrated that non-neuronal cells, including astrocytes and microglia, can have a crucial role in both idiopathic and inherited forms of the disease [77,87,95]. Additionally, mitochondrial dysfunction in neurons, astrocytes and microglia may have a devastating impact on the function and survival of these cells, hence on overall brain homeostasis. Thus, understanding the molecular mechanism regulated by PINK1 in the brain will aid in gaining knowledge on how overall mitochondrial homeostasis is underlying several PD pathologies.
